# In vivo monitoring of lung inflammation in CFTR-deficient mice

**DOI:** 10.1186/s12967-016-0976-8

**Published:** 2016-07-28

**Authors:** Fabio Stellari, Gabriella Bergamini, Francesca Ruscitti, Angela Sandri, Francesca Ravanetti, Gaetano Donofrio, Federico Boschi, Gino Villetti, Claudio Sorio, Barouk M. Assael, Paola Melotti, Maria M. Lleo

**Affiliations:** 1 Pharmacology & Toxicology Department Corporate Pre-Clinical R&D, Chiesi Farmaceutici, Largo Belloli, 11/A, 43122 Parma, Italy; 2 Dipartimento di Medicina, Università di Verona, Verona, Italy; 3 Dipartimento di Scienze Biomediche, Biotecnologiche e Traslazionali, Università di Parma, Parma, Italy; 4 Dipartimento di Diagnostica e Salute Pubblica, Università di Verona, Verona, Italy; 5 Dipartimento di Scienze Medico Veterinarie, Università di Parma, Parma, Italy; 6 Dipartimento di Informatica, Università di Verona, Verona, Italy; 7 Centro Fibrosi Cistica, Azienda Ospedaliera Universitaria Integrata Verona, Verona, Italy

**Keywords:** In vivo bioluminescence imaging, Lung inflammation mouse model, Pseudomonas aeruginosa, CFTR-deficient mice

## Abstract

**Background:**

Experimentally, lung inflammation in laboratory animals is usually detected by the presence of inflammatory markers, such as immune cells and cytokines, in the bronchoalveolar lavage fluid (BALF) of sacrificed animals. This method, although extensively used, is time, money and animal life consuming, especially when applied to genetically modified animals. Thus a new and more convenient approach, based on in vivo imaging analysis, has been set up to evaluate the inflammatory response in the lung of CFTR-deficient (CF) mice, a murine model of cystic fibrosis.

**Methods:**

Wild type (WT) and CF mice were stimulated with *P. aeruginosa* LPS, TNF-alpha and culture supernatant derived from *P. aeruginosa* (strain VR1). Lung inflammation was detected by measuring bioluminescence in vivo in mice transiently transgenized with a luciferase reporter gene under the control of a bovine IL-8 gene promoter.

**Results:**

Differences in bioluminescence (BLI) signal were revealed by comparing the two types of mice after intratracheal challenge with pro-inflammatory stimuli. BLI increased at 4 h after stimulation with TNF-alpha and at 24 h after administration of LPS and VR1 supernatant in CF mice with respect to untreated animals. The BLI signal was significantly more intense and lasted for longer times in CF animals when compared to WT mice. Analysis of BALF markers: leukocytes, cytokines and histology revealed no significant differences between CF and WT mice.

**Conclusions:**

In vivo gene delivery technology and non-invasive bioluminescent imaging has been successfully adapted to CFTR-deficient mice. Activation of bIL-8 transgene promoter can be monitored by non-invasive BLI imaging in the lung of the same animal and compared longitudinally in both CF or WT mice, after challenge with pro-inflammatory stimuli. The combination of these technologies and the use of CF mice offer the unique opportunity of evaluating the impact of therapies aimed to control inflammation in a CF background.

**Electronic supplementary material:**

The online version of this article (doi:10.1186/s12967-016-0976-8) contains supplementary material, which is available to authorized users.

## Background

In cystic fibrosis (CF), chronic neutrophilic inflammation with the release of damaging neutrophil products, such as neutrophil elastase, constitutes a key risk factor in early structural lung damage and lung function decline [1]. Bacterial infection stimulates an intense neutrophilic response which fails to eradicate the infection leading to sustained release of pro-inflammatory mediators, continuous influx of inflammatory cells and bacteria persistence [2]. Despite advances in our understanding of the molecular and cellular basis of CF, the paradox of why recruited neutrophils fail to eradicate bacterial infections in the lung remains [2, 3], although recent data from our group indicate a deficit in leukocyte trafficking as a possible mechanism [4]. The progress in understanding the relationship between cystic fibrosis airway inflammation and infection may facilitate the understanding on how to interrupt the self-perpetuating inflammatory response and pursue new directions in treatment.

Measuring airway inflammation in CF is important for initiating the anti-inflammatory treatment and monitoring its effect. At present, no inflammatory biomarker has been consistently shown to predict clinical efficacy [3]. Frequently, in mouse models, inflammation is induced by instillation of bacterial products with pro-inflammatory activity such as lipopolysaccharide (LPS) and monitored by analyzing the presence of inflammatory markers. Unfortunately, the conventional assessments of inflammation in mice often rely on invasive ex vivo measurements which causes the death of the animal and are consequently particularly onerous when costly transgenic mice are utilized. Moreover, this protocol may not be fully reproducible, probably due to the heterogeneity of airway inflammation in CF. Furthermore, the characterization of pulmonary functions, cellular infiltration and cytokines determination in BALF and the observation of anatomical changes, such as airway remodeling due to inflammation, an approach that, while extensively used and highly validated, precludes the possibility to repeat longitudinally the assessment of test animals [5–7]. However, in vitro and in vivo protocols in animal models have been used to study the mechanism of lung inflammation in chronic diseases and to evaluate the anti-inflammatory role of some candidate molecules [8–10]. Recently emerging non-invasive imaging technologies such as magnetic resonance imaging (MRI), micro-CT and optical imaging have been applied to longitudinal monitoring of airway remodeling and inflammation in murine models [11–14]. The clinical imaging system used in MRI, CT and PET, have been further adapted to murine models of asthma [11, 15–17], to serve as a preclinical and translational step between basic discovery and clinical practice, whereas optical imaging technologies developed in experimental settings may also slow-down their way into the clinical practice, especially in the context of intraoperative activities [18, 19]. Recently a mouse model transiently expressing the luciferase reporter gene under the control of a bovine IL-8 promoter has been generated [20]. Although mice do not express IL-8, the cell signaling and transcriptional apparatus could specifically activate the bovine IL-8 promoter [20]. In the present work, real time monitoring of lung inflammation in CF mice has been successfully applied by taking advantages of the genetic construct carrying the IL-8 promoter/luciferase reporter gene.

## Methods

### Experimental animals

Gut-corrected CFTR-deficient female C57Bl/6 Cftrtm1UNCTgN(FABPCFTR)#Jaw and their congenic WT female mice, 6–8 weeks old (Charles River), provided by the (CFaCore facility, Milano, Italy), were used. Prior to use, animals were acclimatized for at least 5 days to the local vivarium conditions (room temperature: 20–24 °C; relative humidity: 40–70 %; 12-h light–dark cycle), having free access to standard rodent chow and softened tap water. All animal experiments described herein were approved by the intramural animal-welfare committee for animal experimentation of Chiesi Farmaceutici and by the Interdeparmental Centre of Experimental Research Service at the University of Verona, and comply with the European Directive 2010/63 UE, Italian D.Lgs 26/2014 and the revised “Guide for the Care and Use of Laboratory Animals”.

### Collection of bacterial cell-free supernatants

*P. aeruginosa* strain, VR1 was isolated from a sputum sample of a CF patient followed at the Cystic Fibrosis Center in Verona, Italy. Written informed consent was obtained from the subjects enrolled in the study and approved by the Institutional Review Board of Azienda Ospedaliera Universitaria Integrata (AOUI) Verona as project 1612. The bacterial strain was grown overnight at 37 °C in Bacto™ Tryptic Soy Broth (TSB, Becton, Dickinson and Company) with continuous agitation. The day after, *P. aeruginosa* cells were diluted in TSB to the concentration of 1 × 10^8^ CFU/ml (OD of 0.1 at 600 nm) and grown overnight at 37 °C shaking. The following day, the culture was normalized to an optical density of 0.2 OD at 600 nm. Culture supernatant (VR1Sn) was collected by centrifugation (7000×*g*, 30 min, 4 °C) and filtered through a 0.22 µm Millipore filter to remove any remaining bacteria. VR1Sn was concentrated to 30× using Amicon Ultra-15 30 K (Millipore, Billerica, USA), then centrifuged at 27,000×*g* for 1 h at 4 °C to remove cellular debris and finally sterilized by filtration through a Millipore 0.22 µm filter.

### In vivo gene delivery

The bIL-8-Luc plasmid (Dipartimento di Scienze Medico Veterinarie, Università di Parma, Italy) was obtained by sub-cloning the 2030 bp IL8 bovine promoter, amplified by PCR from Madin–Darby bovine kidney (MDBK; ATCC #CCL-22) genomic DNA into the digested pGL3basic vector (promega) as previously described [20]. We applied in vivo JetPEI (Polyplus Transfection) as a carrier for delivering DNA to lung tissues. The DNA and JetPEI mix was formulated according to the product manual with a final N/P ratio of 7.5. Briefly, 50 μg of bIL-8–Luc and 7.5 μL of JetPEI were both diluted into 200 μL 5 % glucose. The two solutions were then mixed and incubated for 15 min at room temperature. The entire mixture was injected intravenously in mice and the expression of bIL-8–Luc was monitored through imaging with IVIS Spectrum (Perkin Elmer, Massachusetts, USA).

### In vivo bioluminescence imaging

Transfection per se causes a mild lung inflammatory response and bIL-8 activation, which is detectable by BLI up to 3–4 days after DNA injection and disappears completely after 1 week [20, 21]. No difference in extension and duration of BLI signal between wild type and CF mice at 3–4 and 7 days of observation after DNA delivery has been observed. (Additional file 1: Figure S1). One week after DNA delivery, before initiating the experiment, all the transient transgenic mice were injected intraperitoneally (i.p.) with luciferin (150 mg/kg) and BLI was recorded in order to check the baseline activation of the IL-8 pathway. Slightly differences in promoter activation detected in the different mice can be normalized by dividing the BLI value obtained from each mouse with the basal photon emission found in the same mouse in order to determine the fold of induction (FOI) as presented in the graphs. This procedure ensures that the data are referred to each individual animal and take in account even slightly different response rates.

Briefly, 10 and 15 min after luciferin injection, mice were lightly anesthetized with isoflurane and images were obtained using the IVIS imaging system: a total of photons emitted from the chest of the mice was quantified using Living Image ^®^ software (Perkin Elmer Inc. Boston, MA, USA).

The following day, mice were intratracheally challenged with *P. aeruginosa* LPS (6.125 µg/mouse), VR1Sn (10×) or human TNF-alpha (1 µg/mouse) using a volume of 50 μl and BLI was recorded at 4, 24, and 48 h. Data were expressed as mean folds of induction (FOI) over the baseline activation of each mouse.

### Bronchoalveolar lavage and cytokines

Twenty-four hours after intratracheal challenge, animals were weighted, anaesthetized with isoflurane and sacrificed by bleeding from the abdominal aorta for bronchoalveolar lavage fluid (BALF) collection, performed as previously described [20]. BALF supernatants were frozen at −80 °C for simultaneous quantification of multiple cytokines/chemokines using a Bio-Plex™ Cytokine Assay Kit (Bio-Rad Laboratories, Segrate, Milan, Italy). The cell pellet was resuspended in 0.2 mL of phosphate buffered saline (PBS) and total and differential cell counts were obtained using an automated cell counter (Dasit XT 1800 J, Cornaredo; Milan, Italy).

### Histology

Lungs were removed and inflated with a cannula through the trachea by gentle infusion with 0.6 ml of 10 % neutral-buffered formalin. Subsequently, the lungs were placed in 10 % formalin and fixed for at least 24 h. The whole lungs were dehydratated in graded ethanol series, clarified in xylene and paraffin embedded. 5 μm thick sections were cut with a rotary microtome (Reichert–Jung 1150/Autocut) in ventral dorsal plane. For analysis, the sections were stained with hematoxylin-eosin and Giemsa according to standard methods. A previously described lung injury score [22], that included the evaluation of neutrophils in the alveolar and in the interstitial space, the formation of hyaline membranes, the presence of proteinaceus debris and the alveolar septal thickening was applied.

### Reagents

In vivo JetPEI DNA transfection reagent (polyplus transfection) was obtained from Euroclone (Milan, Italy), D-luciferin was obtained from Perkin Elmer Inc. (Boston, MA, USA). LPS (from *P. aeruginosa* 0111:B4, product n.L3012) from Sigma (St. Louis, MO) was resuspended in Eagle’s minimal essential medium (EMEM).

### Data analysis

Experimental values were expressed as the mean and standard error of the mean (SEM). Statistical analysis was performed using one-way analysis of variance followed by Dunnett’s t test (*p < 0.05, **p < 0.01).

## Results

**Fig. 1:**
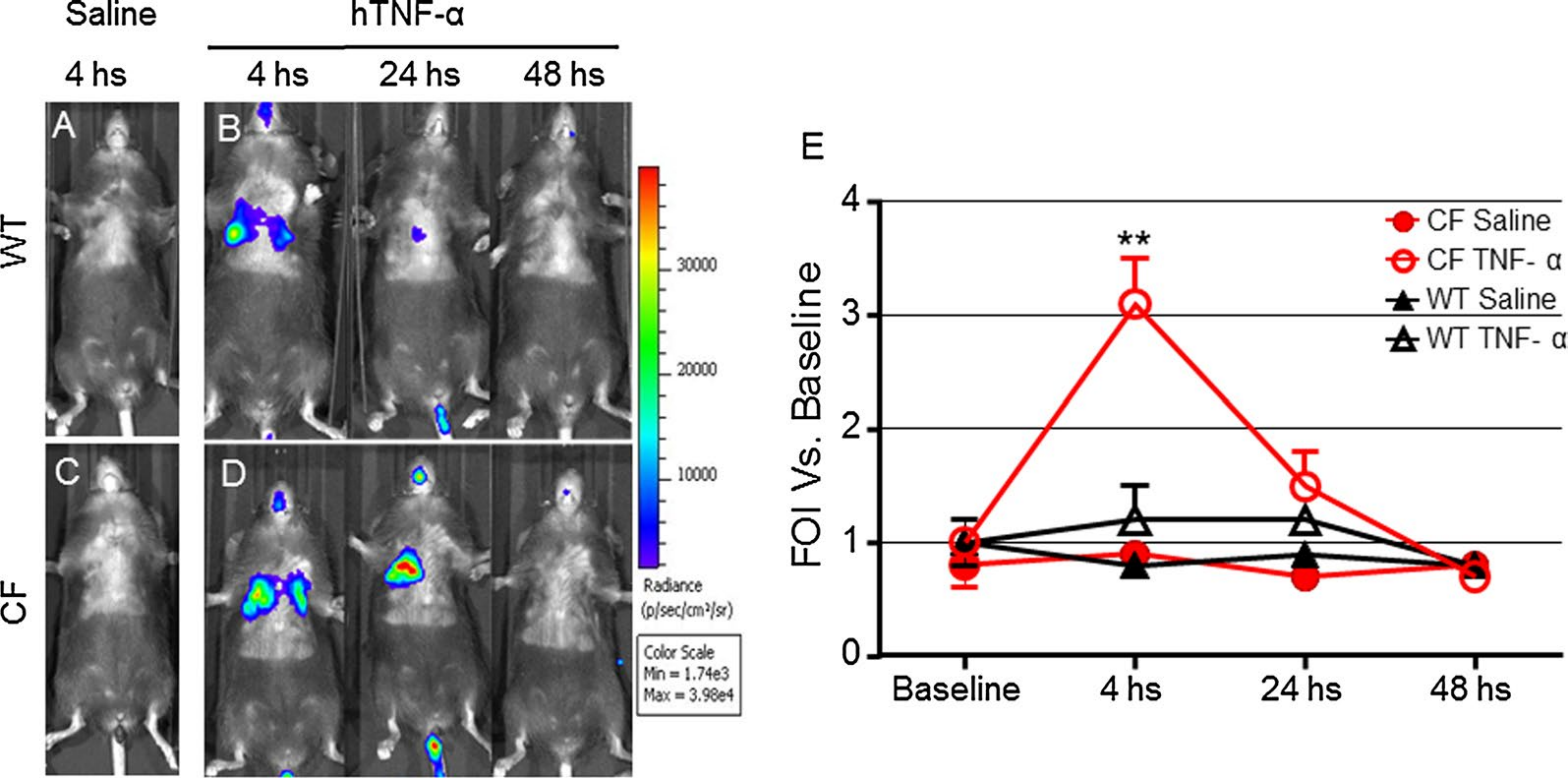
Representative in vivo images of WT and CF mice transiently transgenized with bIL-8-Luc and intratracheally challenged with hTNF-alpha (1 µg/mouse) and monitoring of bIL-8 activation by BLI. **a, c**: WT and CF mice treated with saline solution (control). **b, d**: WT and CF mice after intratracheal instillation with hTNF-alpha and monitoring of IL-8 activation at 4, 24 and 48 h by BLI. **e**: Time-course of IL-8-Luc activation in WT and CF mice. Results are reported as folds of increase (FOI) over baseline as mean ± SEM, n = 9 each group. Statistical differences were tested by one-way ANOVA followed by Dunnett’s t post hoc test for group comparisons. *p < 0.05 and **p < 0.01

**Fig. 2:**
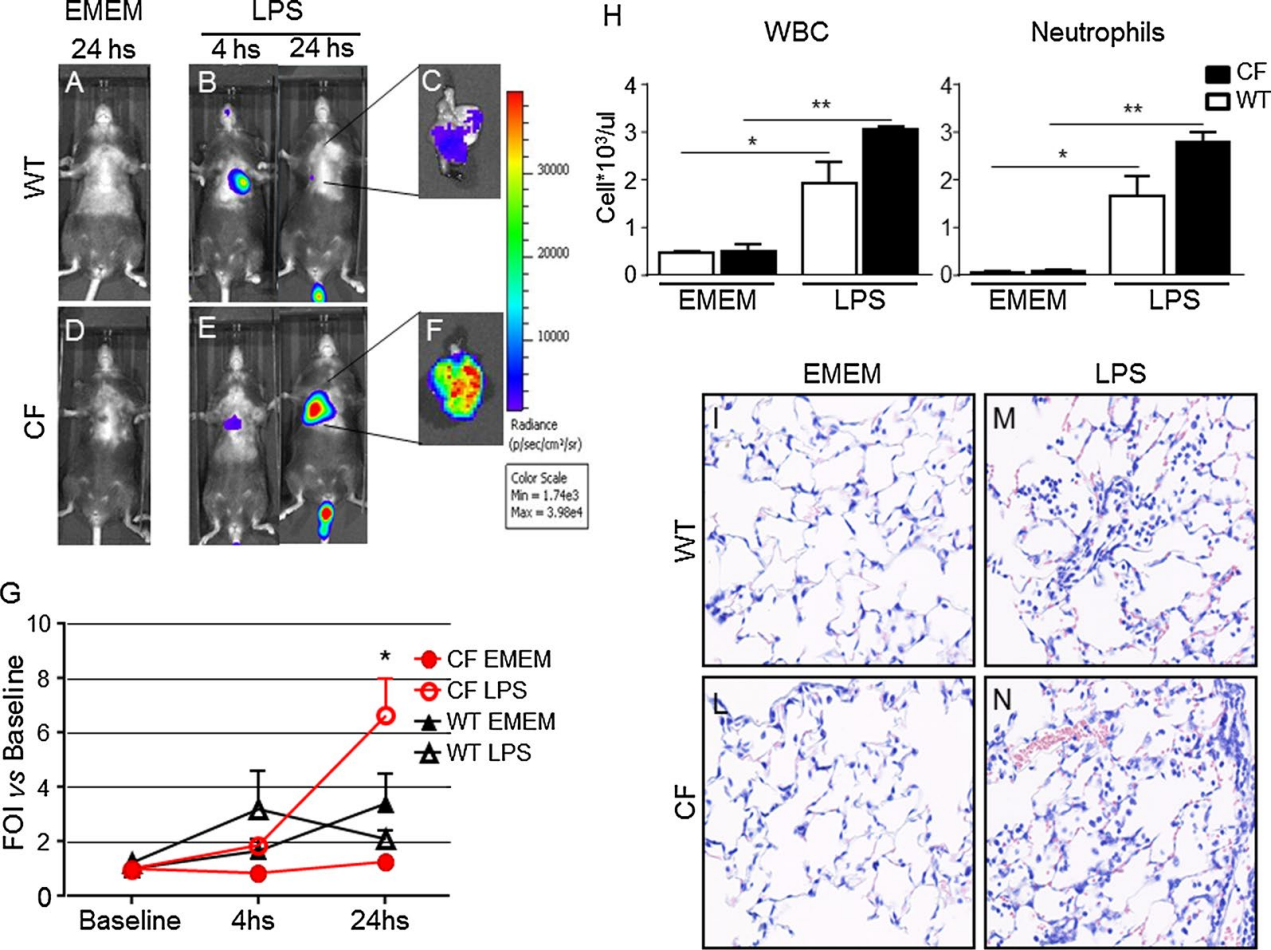
Representative in vivo images of WT and CF mice transiently transgenized with bIL-8-Luc and intratracheally treated with *Pseudomonas* LPS (6.25 µg/mouse). **a, d**: WT and CF mice treated with EMEM (control). **b, e**: WT and CF mice after intratracheal instillation with LPS and monitored IL-8 activation at 4 and 24 h by BLI. **c, f**: ex vivo images of IL-8 activation in lungs excised from WT and CF mice at 24 hs after LPS challenge. *Panel G*: Time-course of IL-8-luc activation in WT and CF mice. Results are reported as folds of increase (FOI) over baseline as mean ± SEM, n = 6 at each group. **h**: Cellular infiltration into the lung of WT and CF mice after intratracheal instillation with LPS at 24 h. Three mice were used for every time point and the experiment was replicated three times. Values are expressed as mean ± SEM of the three different experiments. Statistical differences were tested by one-way ANOVA followed by Dunnett’s t post hoc test for group comparisons. *p < 0.05 and **p < 0.01 WT and CF TSB vs WT and CF LPS group. (**I**–**n**): Representative photomicrographs of Giemsa stained lung of WT and CF mice after intratracheal instillation with EMEM (I, L) and LPS (M, N) at 24 h. Magnification 40×

**Fig. 3:**
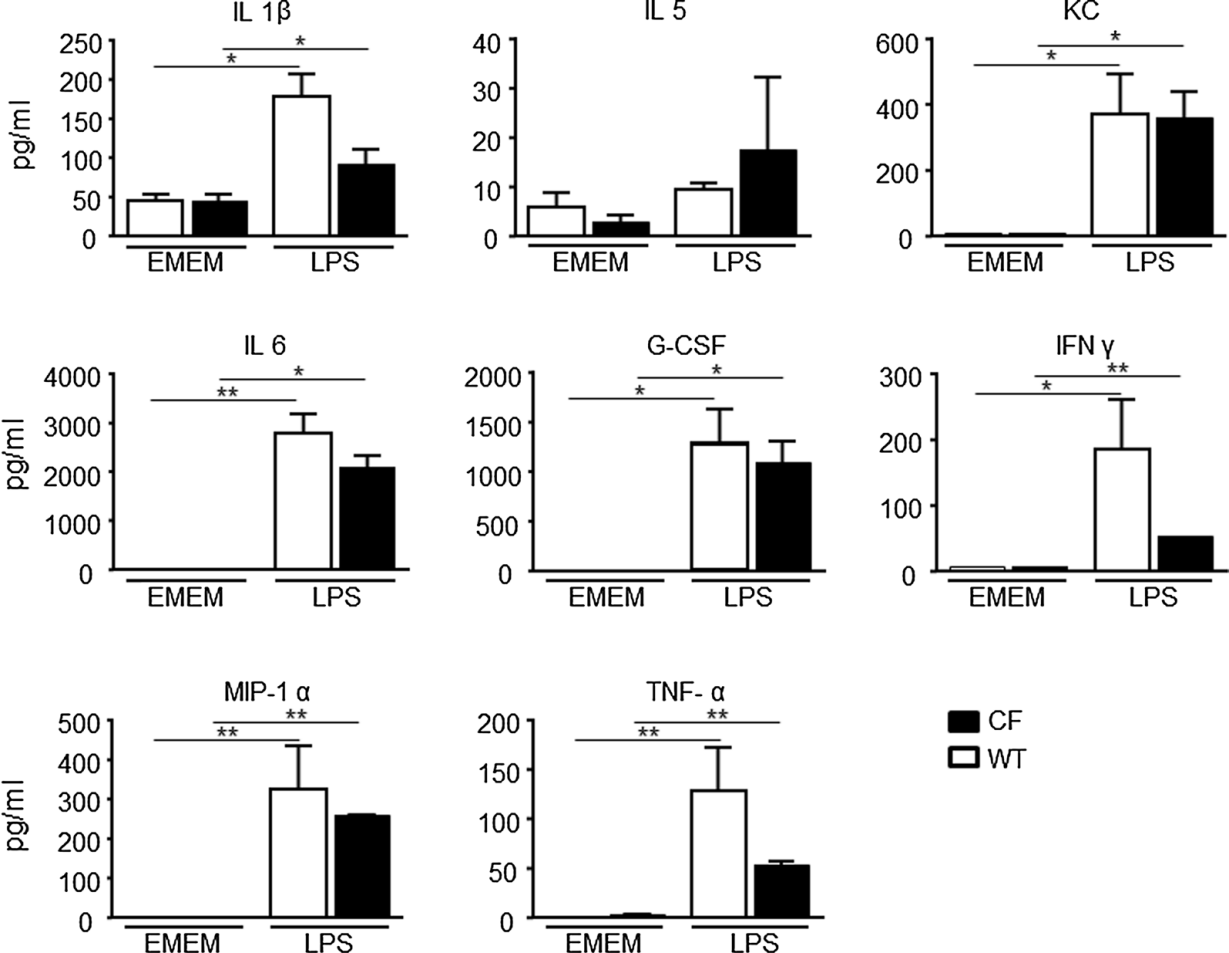
Cytokines quantification in BALFs. WT and CF mice, treated with either EMEM or LPS and sacrificed at 24 h. A panel of 23 cytokines was analysed using a Bio-Plex™ Cytokine Assay Kit (Bio-Rad Laboratories). Eight resulted up-regulated compared to the control animals: IL-1β, IL-5, KC, IL-6, G-CSF, INFγ, MIP-1α, and TNF-α Three mice were used for every time point and the experiment was replicated three times. Values are expressed as mean ± SEM. Statistical differences were tested by one-way ANOVA followed by Dunnett’s t post hoc test for group comparisons. *p < 0.05 and **p < 0.01

The possibility to monitor longitudinally in no-invasive way the activation of IL-8 in the same mice CF compared to WT after intratracheally challenged with inflammatory stimuli such as: human TNF-alpha, LPS P. aeruginosis and with the culture supernatant obtained from the *P. aeruginosa* clinical strain VR1 is step a forward in investigating the molecular mechanism linked to cystic fibrosis and with the advantage of reducing the number of animals required. Starting from the previously acquired assumption that intratracheal instillation of hTNF-alpha 1 week after delivery of the luciferase reporter DNA construct caused activation of bIL-8 in mice that could be easily detected and monitored by IVIS [20]; it was of interest to know if the same system could be applied in CF mice. To this end, CF mice were transiently transgenized with bIL-8-Luc.DNA construct, intratracheally challenged with hTNF-alpha (1 µg/mouse) and compared with WT mice (Fig. 1) Luc expression driven by bIL-8 activation at 4 h after TNF-alpha challenge (Fig. 1e) revealed a significant increase of BLI in CF mice in comparison with WT animals (3.1 and 1.2 FOI, p < 0.01) over baseline (untreated mice) (Fig. 1b, d, e). The BLI signal was still detectable without significant differences at 24 h (Fig. 1b, d) in both CF and WT mice (1.5 and 1.2, FOI respectively), while at 48 h the BLI signal was not detectable anymore. CF and WT mice intratracheally treated with saline did not show any BLI signal either at 4 h (Fig. 1a, c) or at later time points of observation. This preliminary set of experiments allowed us to select the optimal transfection conditions and to determine the right time of imaging acquisition. Although the gene delivery technology has been well established [20, 21, 23, 24], every strain of mouse required their own transfection conditions and responded to stimuli in a different manner. C57Bl/6 mice have a black fur that is an obstacle for optical imaging technologies, because black color quenched partially the light even if they were shaved in order to minimize the interference with BLI signal. In the next series of experiments, imaging has been performed only at 4 and 24 h because the BLI signal significantly decreased at 48 h. The same mice challenged with hTNF-alpha, have been reutilized and intratracheally challenged with *P. aeruginosa* LPS (6.25 µg/mouse) after 2 weeks of wash-out. As shown in Fig. 2, both CF and WT mice responded to LPS reaching the maximum BLI signal between 4 and 24 h post-challenge. Interestingly, WT animals challenged with *P. aeruginosa* LPS showed a higher BLI signal compared to CF mice already at 4 h (3.2 and 1.9 FOI respectively) over baseline (Fig. 2b, e, g). However only at 24 h, the BLI signal was significantly higher in CF compared to WT mice (6.7 and 2.1 FOI, p < *0.05*) (Fig. 2b, e, g). No significant increases in the BLI signal was detected when CF and WT mice were intratracheally treated with the culture medium (EMEM) (Fig. 2a, d). After imaging at 24 h, the same mice were sacrificed for BALFs collection and histological analysis. Right after BALF sampling, lungs were excised and imaging was performed. As clearly shown in ex vivo pictures the BLI signal was well localized in the lung and higher in CF compared to WT mice (Fig. 2c, f), lending support to the positive correlation between in vivo and ex vivo imaging. Formally, information collected by BLI (bioluminescent signal in the lung higher in CF compared to WT mice) and from BALF (WBC and neutrophils counts in BALFs in both mouse strains) cannot directly compared and put “in contrast” to each other. (Fig. 2h). The lungs of CF and WT challenged with either EMEM (Fig. 2i, m) or *P. aeruginosa* LPS (Fig. 2l, n) were fixed in formalin for histological analysis after Giemsa staining. As expected, *P. aeruginosa* LPS-induced lung injury was characterized by neutrophils accumulation, alveolar wall thickening and proteinaceous detritus accumulation in the alveolar space of the lungs of either CF or WT mice when compared to control mice treated with EMEM. Area of emphysema and a slight degree of fibrosis were detected within the lung parenchyma of WT and CF mice without any significant differences as regards the type and degree of histological damage (Fig. 2m, n). Eight in 23 cytokines evaluated in BALFs of WT and CF mice 24 h after stimulation with *P. aeruginosa* LPS, resulted up-regulated when compared to the control animals (Fig. 3). IL-1β, IL-5, KC, IL-6, G-CSF, INFγ, MIP-1α, and TNF-α, are the major pro-inflammatory mediators in acute immune-response and they increased in the same manner in CF and WT LPS challenged mice. Their up-regulation resulted in full agreement between inflammatory cells infiltration and histological analysis (Fig. 2h, m, n).

**Fig. 4:**
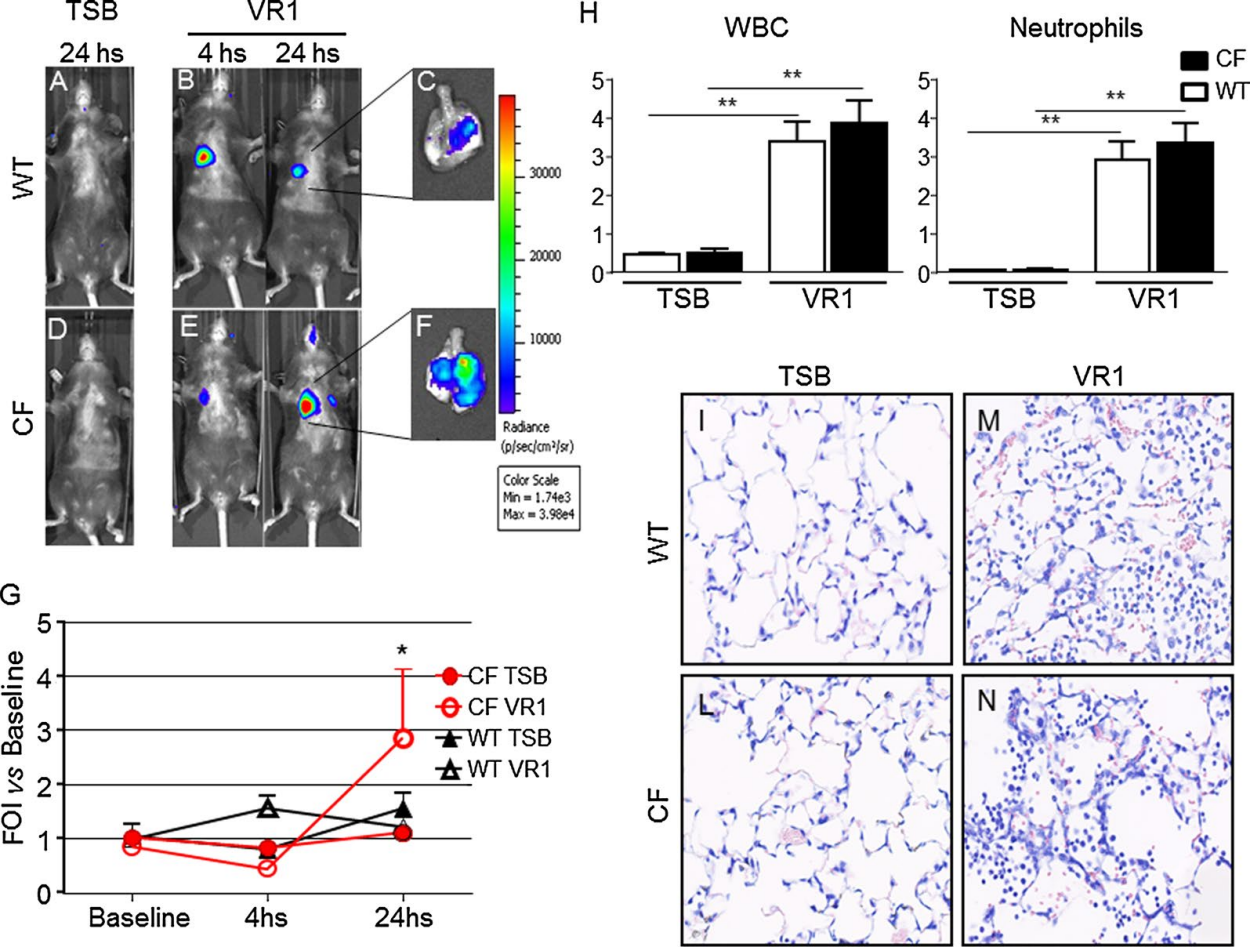
Representative in vivo images of WT and CF mice transiently transgenized with bIL-8-Luc intratracheally challenged with VR1 culture supernatant (10X/mouse), and monitoring of bIL-8 activation by BLI. **a**, **d**: WT and CF mice after intratracheal instillation with TSB (control). Panel B, E: WT and CF mice after intratracheal instillation with VR1Sn and monitoring of IL-8 activation at 4 and 24 h by BLI. **c**, **f**: ex vivo images of IL-8 activation in lungs excised from WT and CF mice at 24 h. **g**: Time-course of IL-8-luc activation in WT and CF mice after challenge with intratracheal VR1Sn. Results are reported as FOI over baseline as mean ± SEM, n = 6 for each group. *Panel H*: Cellular infiltration into the lung of WT and CF mice after intratracheal instillation with VR1Sn. VR1Sn-induced neutrophils and white blood cells (WBC) recruitment in the airways at 24 h. Three mice were used for every time point and the experiment was replicated three times. Values are expressed as mean ± SEM of the three different experiments. Statistical differences were tested by one-way ANOVA followed by Dunnett’s t post hoc test for group comparisons. *p < 0.05 and **p < 0.01 WT and CF TSB vs WT and CF VR1 group. (**i**–**n**) : Representative micro-photographs Giemsa staining of lung of WT and CF mice after intratracheal instillation with TSB(I, L), and VR1Sn (M, N) at 24 h. Magnification 40×

**Fig. 5:**
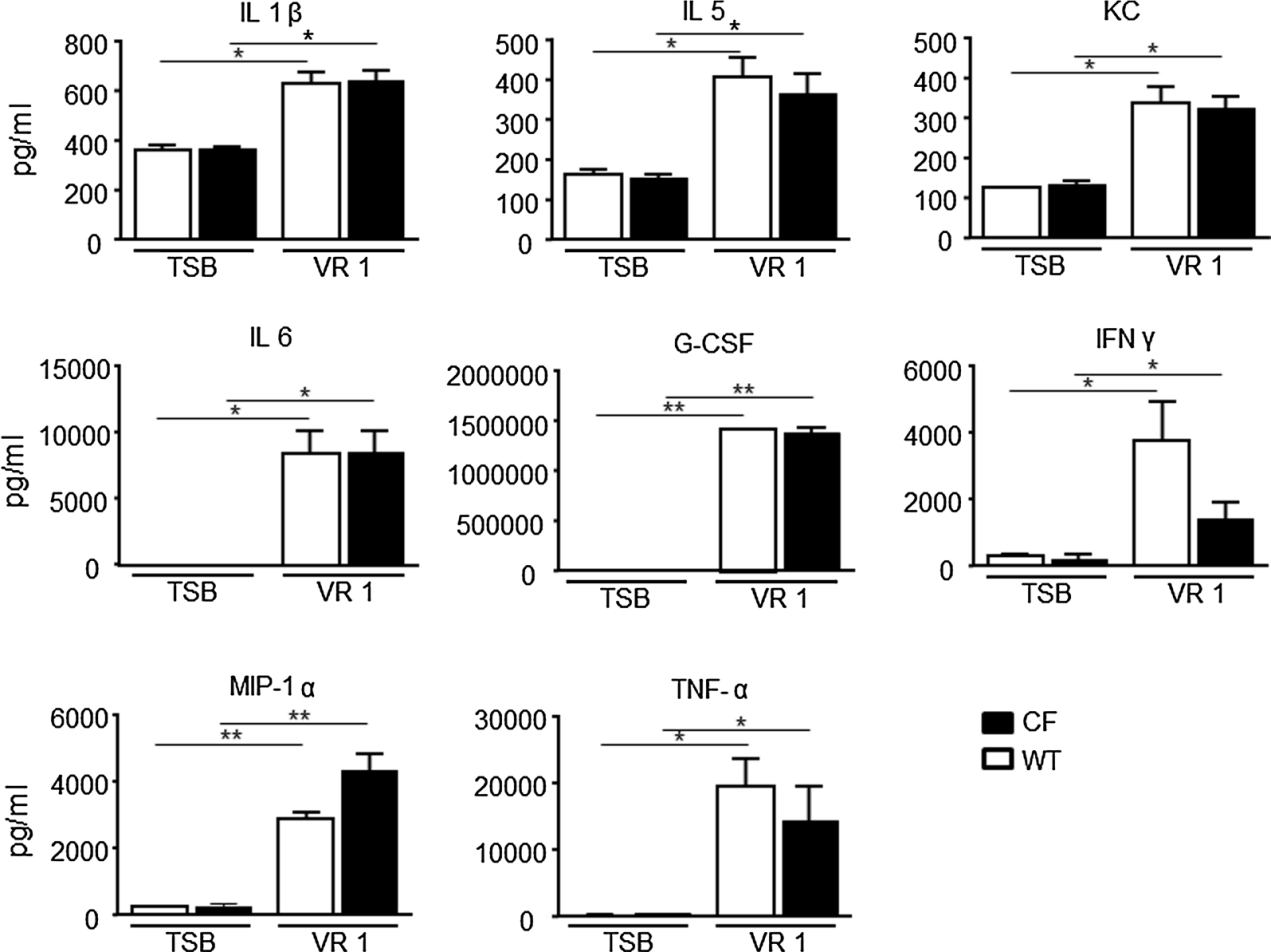
Cytokines quantification in BALFs. WT and CF mice, treated with either TSB or VR1Sn and sacrificed at 24 h. A panel of 23 cytokines was analysed using a Bio-Plex™ Cytokine Assay Kit (Bio-Rad Laboratories), and eight resulted up-regulated compared to the control animals: IL-1β, IL-5, KC, IL-6, G-CSF, INFγ, MIP-1α, and TNF-α Three mice were used for every time point and the experiment was replicated three times. Values are expressed as mean ± SEM. Statistical differences were tested by one-way ANOVA followed by Dunnett’s t post hoc test for group comparisons. *p < 0.05 and **p < 0.01

In another series of experiments, WT and CF mice were challenged with the supernatant culture (10X) obtained from the *P. aeruginosa* clinical strain VR1 [25]. As recently reported [25], this strain produces and releases in the culture medium a number of virulence factors, such as metalloproteases, pyocianine and pyoverdine, showing pro-inflammatory activity. Intratracheally treatment with VR1Sn was able to activate bIL-8 in transiently transgenic CF and WT mice. (Fig. 4g). WT animals responded with an higher BLI signal at 4 h (1.6 FOI) compared to CF mice, which revealed a very weak signal intensity at the same time point of observation (Fig. 4b, e, g). Nevertheless 24 h after challenge, BLI signal increased significantly in CF compared to WT mice (2.9 and 1.2 FOI, p < *0.05*) (Fig. 2b, e, g). No significant increases in the BLI signal was detected when CF and WT mice when intratracheally treated with TSB (Fig. 2a, d). Either *P. aeruginosa* LPS or VR1Sn showed a similar behavior on bIL-8 activation. WT animals responded with max BLI signal at 4 h, indicating an early activation of the gene while, on the contrary, CF mice reveled a late response at 24 h. After in vivo imaging at 24 h, mice previously imaged were sacrificed, BALFs collected and lungs removed and subjected to histological analysis and BLI imaging as clearly shown in ex vivo pictures (Fig. 4c, f). The BLI signal was higher in CF confirming the in vivo bioluminescence imaging data. Surprisingly and in contrast with BLI signal, VR1Sn induced an increase in total WBC and neutrophils in BALFs of CF and WT mice, but significant differences were not detectable (Fig. 4h). Lungs coming from TSB or VR1Sn challenged WT and CF mice were fixed in formalin and a Giemsa staining was performed. A widely diffused mononuclear cell infiltrate characterized all the VR1Sn-challenged samples either from CF or WT mice as compared to control mice in which a normal parenchyma was presented (Fig. 4i, l). Histologically, the alveolar space and thicker alveolar septa were filled with acute phase inflammatory cells, including neutrophils, eosinophils, alveolar macrophages and lymphocytes. Within the alveolar space also proteinaceous detritus were detected. However, the same type and severity of histological damage was observed in WT and CF mice (Fig. 4m, n). Up-regulation of the same set of cytokines after *P. aeruginosa* LPS and VR1 culture supernatant treatment was observed (Fig. 5).

## Discussion

The animal model previously developed has been successfully adapted to CFTR-deficient mice. After induction with human TNF-alpha and *P. aeruginosa* LPS, it was possible to detect BLI signal exactly from the thorax areas with higher signals in CF animals with respect to WT mice at 24 h after challenge. Bioluminescent signal was still detectable for as long as 48 h, although at this time point the signal decreased and it became difficult to appreciate the differences between the two types of mice. Further validation by challenging the mice with culture supernatants containing virulence factors released by a clinical strain of *P. aeruginosa* previously characterized [25]. Also in this case, statistically significant differences between WT and CF inflammatory responses were revealed with a stronger response of CF mice as compared to WT animals at 24 h. Lung inflammatory markers, such as immune cells, cytokines and histology, were monitored in BALF after pro-inflammatory stimulation but they did not show significant differences between CF and WT mice. Although surprising at a first glance, we must consider that a differential degree of strain sensitivity to pro-inflammatory stimuli has been reported [26]. Indeed, the same intratracheal challenge consisting in VR1Sn (10X) was used in BALB/C mice and the absolute number of inflammatory cells recruited into the lung 24 h after treatment was much higher than in the C57Bl/6 mouse strain [25], supporting this interpretation. The reporter gene utilized relies on bIL-8 promoter, which responds mainly to NF-κB while additional master genes, like AP-1 (Fos/Jun), NFATs, and STATs, control the expression of several cytokines [27]. Such additional pathways can be activated in different mouse strains that might bypass the need of full NF-κB recruitment. In our specific case, being the C57Bl/6 mice congenic, the differential response to agonists further highlight the critical role of CF background in controlling inflammatory response.

At the moment we have not an explanation to the fact that we detected a stronger BLI signal in CF mice as compared to WT animals although the immune response, measured with classical BALF markers, was comparable in the two type of mice. However, this undoubtedly might constitute an advantage allowing the detection of CF-specific differences in the inflammatory response also in those cases in which both WT and CF responded equally as regards cell recruitment, cytokines expression and histological changes.

Despite the fact that CFTR-deficient mouse model is considered of limited interest in the field of cystic fibrosis as it does not reproduce and develop the full CF phenotype [28]. Our data support the notion that differential responses in CFTR defective-mice may occur, a fact that is in line with the pro-inflammatory phenotypes recognized in humans.

## Conclusions

Non-invasive imaging techniques are increasingly considered in biomedical research and can lead to important insights on disease development on the same mouse model. In this study we combined in vivo gene delivery technology and non-invasive bioluminescence imaging, creating a unique tool to consider CF mice as suitable models in testing anti-inflammatory drugs and in investigating the molecular mechanism linked to cystic fibrosis [4]. Moreover, the possibility to repetitively test a single set of CF animals longitudinally has several advantages as it reduces the number of animals to sacrifice and the costs of housing and increases the data quality by lowering the intra animals variability, since each mouse represent its own control. This principle, applied to a CFTR-deficient murine model, will permit to study the activation of the inflammatory pathways in the CFTR defective lung in response to different agonists and to study/identify bacterial products with pro-inflammatory activity. Furthermore, the model might be used to evaluate, in an in vivo setting, the possible anti-inflammatory effect of drugs and therapeutic treatments in a cystic fibrosis background.

## Additional file

10.1186/s12967-016-0976-8 In vivo bioluminescence imaging. Monitoring bIL-8 activation in WT and CF transiently transgenized mice with bIL-8-Luc plasmid at 3, 4 and 7 days after DNA delivery. Results are reported photons/sec/cm2 as mean ± SEM, n = 6 each group. Statistical differences were tested by one-way ANOVA followed by Dunnett’s t post hoc test for group comparisons. *p < 0.05 and **p < 0.01.

## Abbreviations

BLI: bioluminescent imaging
BALFs: bronchoalveolar lavage fluids
CF: CFTR-deficient mice
WT: wild type mice
