# Supplementary figures and images for: In vivo monitoring of lung inflammation in CFTR-deficient mice
Source: J Transl Med. 2016 Jul 28;14:226. doi: 10.1186/s12967-016-0976-8 (PMC4964274; doi:10.1186/s12967-016-0976-8)

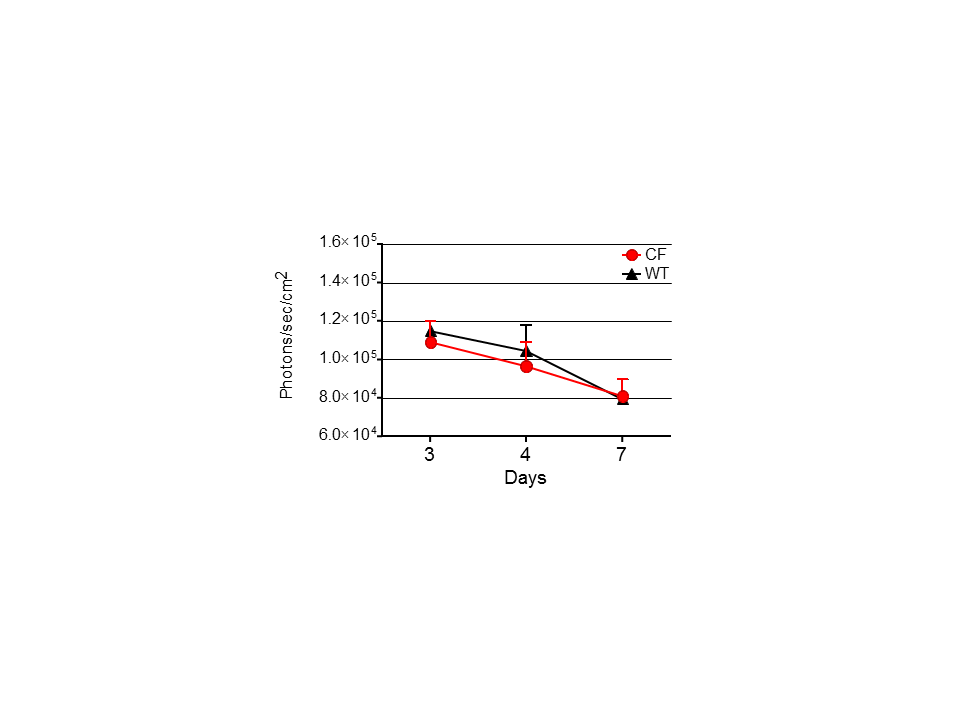

Supplement: Supplementary file 1 — 10.1186/s12967-016-0976-8 In vivo bioluminescence imaging. Monitoring bIL-8 activation in WT and CF transiently transgenized mice with bIL-8-Luc plasmid at 3, 4 and 7 days after DNA delivery. Results are reported photons/sec/cm2 as mean ± SEM, n = 6 each group. Statistical differences were tested by one-way ANOVA followed by Dunnett’s t post hoc test for group comparisons. *p < 0.05 and **p < 0.01. [file 12967_2016_976_MOESM1_ESM.tiff]
